# Age and grit in prioritising intensive care: - a mixed-methods approach of normative challenges

**DOI:** 10.1186/s12910-025-01305-2

**Published:** 2025-10-27

**Authors:** Mia Svantesson, Jarl Gustav, Falk Wiebke, Sandman Lars

**Affiliations:** 1 University Health Care Research Center, Department of Anaesthesiology and Intensive care, Faculty of Medicine and Health, Örebro University, Örebro, Sweden; 2 University Health Care Research Center, Department of Prosthetics and Orthotics, Faculty of Medicine and Health, Örebro University, Örebro, Sweden; 3 Department of Anaesthesiology and Intensive care, Faculty of Medicine and Health, Örebro University, Örebro, Sweden; 4 Department of Medical and Health Sciences, Linköping University, Linköping, Sweden; 5 Region Örebro län, Box 1613, Örebro, 701 16 Sweden

## Abstract

**Background:**

Intensive care unit (ICU) admissions among older patients are increasing, posing significant challenges to already strained healthcare systems. Decision-making around ICU admission in times of limited resources may provide important knowledge about difficult prioritisations, particularly for older patients. Thus, the aim was to investigate ICU-admission decisions for older patients with COVID-19.

**Methods:**

A mixed-methods approach. We audio-recorded ten COVID Rounds and nine Moral Case Deliberations for 34 patients across three Swedish hospitals during the pandemic, and collected data from medical records of 329 patients aged ≥ 65 diagnosed with COVID-19. Data were analysed using qualitative content analysis and multiple regression.

**Results:**

Among 239 patients with documented decisions in medical records, 56% included explicit justifications. The justifications included considerations of medical benefit (not-too- ill/too-ill), general condition (good/frail), age (not-too-old/high age), professional duty (benefit of the doubt/do no harm) and “worth giving it a go” (grit and will to live/lack of will and coping). A minority (31%) of decisions *favoured* ICU admission. Justifications supporting admission were predominantly drawn from discussions in COVID Rounds and MCDs, where patient grit was a recurring argument. In regression analyses, age ≥ 80 years was the only factor significantly associated with not being admitted to ICU and having a documented justification. Few decisions explicitly referred to COVID-19-specific factors.

**Conclusion:**

Our findings reflect patterns similar to pre-pandemic ICU decision-making, suggesting continuity in clinical reasoning. However, the limited documentation of justifications—especially *in favour* of admission—warrants attention, emphasising the need for clearer reasoning in medical records. Our findings identify chronological age as a key triage factor, normatively supported by the ethical principles of non-maleficence, justice, and Sweden’s legal priority-setting principle of Needs and Solidarity—which emphasises care only when benefit is likely. We therefore advocate for national (and potentially international) guidance on triage systems that support a palliative approach for very old patients. While grit may be relevant to ICU admission due to its link to potential benefit, its use raises ethical concerns, particularly in relation to Needs and Solidarity and Human Dignity. We recommend its cautious application pending further research.

## Introduction

 Intensive care unit (ICU) admissions among older patients are increasing, posing significant challenges to already strained healthcare systems [1, 2]. Resources must be prioritised, but the challenge is to find balance between equity and respect for autonomy as well as between beneficence and obligation to avoid causing harm. Particularly factors of age and social function may raise difficult questions about what equity means. It is vital that these values are not only clearly defined but also legitimate within the healthcare system. Research indicates that difficult choices can expose underlying, sometimes controversial values that may need further scrutiny [3], values less visible when resources are more abundant. The COVID-19 pandemic offers a “natural experiment” to uncover these underlying values and principles behind priorities.

Pre-COVID reviews have identified disease severity, comorbidities, functional status, age and bed availability as key factors in ICU triage [4–7]. Age has been viewed as the most important factor [8, 9]. In COVID-19 pandemic guidelines [10–15], comorbidities [16] and frailty were considered important factors regarding older patients [15, 17, 18]. The Swedish guideline for ICU-priorities during the pandemic [19] was criticised for age discrimination and lacking legal grounds when using life-expectancy based on biological age as a criterion [20, 21].

In Swedish studies conducted prior to the COVID-19 pandemic, very old patients (≥ 80 years) were more likely to receive treatment limitations compared to patients aged 65–79 years, despite having similar severity of illness and comorbidities [22, 23]. However, transparent and well-documented decision-making regarding ICU admission remain limited [5, 22, 24]. To better understand the rationale behind such decisions, there is a need to complement medical record data with richer, more nuanced sources—such as audio-recorded ward rounds and ethical reflections on retrospective cases (Moral Case Deliberation). Thus, the aim was to investigate level-of-care decisions for older patients with COVID-19 with focus on justifications and influencing factors. *Research questions*: What was the prevalence of documentation of decisions and justifications? How were the justifications described and how did they differ between multiple data sources? Which justifications were explicitly associated with the COVID-19 pandemic? What factors influenced documentation of decisions, levels-of-care^1^ and justifications?

*Normative question*: Should age and grit matter in ICU admission decision-making?

## Methods

### Design

We applied a mixed methods design, integrating qualitative and quantitative approaches to generate corroborated evidence through triangulation [25].

### Setting

The setting was three hospitals in a county in central Sweden during the COVID-19 pandemic. COVID Rounds were implemented during the first wave, and Moral Case Deliberations (MCD) took place during the second wave. COVID Rounds were a temporary routine in which intensivists provided pre-emptive advice on COVID-wards. MCD is a recognised method of facilitator-led collective ethical inquiry into complex patient cases [26]. In this context, MCD was simplified and focused on retrospective decision-making [27]. The process involved describing the decision-making situation, formulating the ethical question, listing reasons for and against ICU admission, identifying the values at stake, and ultimately making a judgement about the level-of-care, supported by the justification(s) deemed to carry the most moral weight [27, 28].

### Subjects and data-collection

#### Medical records

We collected data from the medical records regarding patients aged ≥ 65 with a primary diagnosis of COVID-19 in the first and second wave of the pandemic. Documented decisions and notes about level-of-care as well as justifications were copied verbatim into the software NVivo for qualitative analysis. Collected quantitative variables included age, sex, comorbidity, frailty, ICU-admission (or not) and level-of-care. For comorbidities, we used the age-adjusted Charlson Comorbidity Index (CCI) scale [16] and for frailty, we used the Clinical Frailty Scale (CFS) [17]. Scores were possible to calculate for all patients regarding CCI, and for 87% of the patients regarding CFS.

### Audio-recording of COVID rounds and moral case deliberations

Intensivists and ward doctors participating in COVID Rounds and MCDs for 34 patients were audio-recorded. The COVID Rounds were conducted on infection, medical, and surgical wards that had been converted into COVID-wards. The MCDs took place in conference rooms within the anaesthesia and intensive care department, where participants gathered around a table, with a screen displaying the MCD steps and, at times, medical record notes.

### Follow-up interviews

Where possible, follow-up questions were asked after the Moral Case Deliberations (MCDs). The open-ended questions focused on participants’ experiences of the discussions and their views on the justifications for the decisions made (no interview guide). Additionally, probes were used based on the audio recordings, such as, “*You said… can you elaborate?”.* The audio recordings were transcribed verbatim.

### Analysis

#### Descriptive statistics

**Fig. 1:**
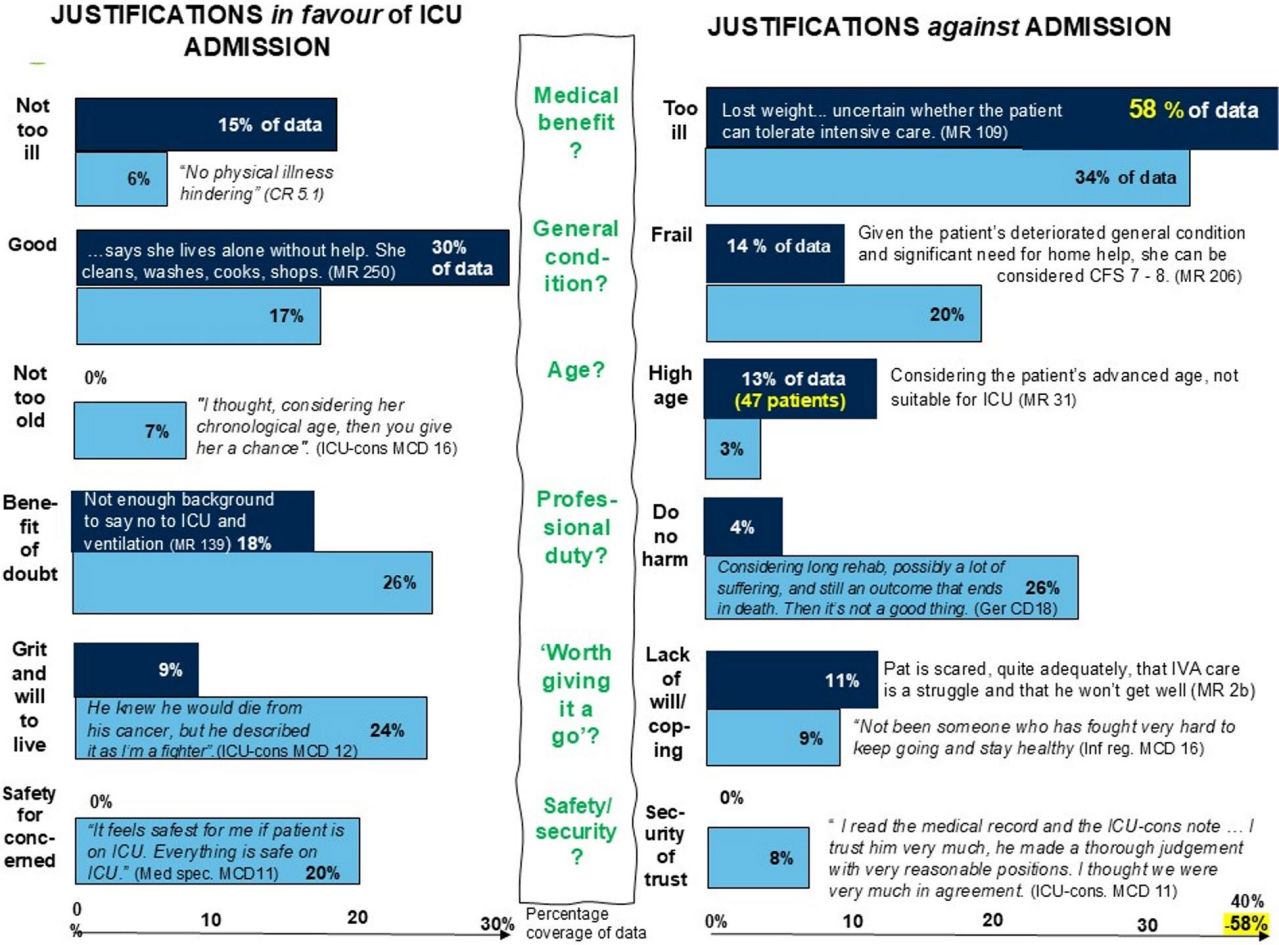
Percentage text coverage of justifications *in favour of* and *against* ICU, in medical records (MR) and transcribed data from COVID rounds (CR) and Moral Case Deliberations (MCD)

First, we calculated the frequency of documented level-of-care decisions and frequency of justifications of these decisions, in the medical records. Second, frequencies of the qualitative analysis described below, that is, condensed meaning-units of justifications for the level-of-care decisions were calculated through the NVivo program [29] as well as the percentage coverage of the transcribed text and medical records notes (Fig. 1). Quantifying the qualitative findings is in line with phenomenography giving weight to recurring topics [30].

### Qualitative analysis

We applied content analysis [31] to the medical record notes and transcribed audio-recordings, adapted for the use of the software NVivo v.12 [32]. This comprised iterative reciprocal actions of coding, moving and reformulating condensed meaning-units and categories on different abstraction levels in an emerging understanding between the whole and the parts (see Table 1).

**Table 1:**
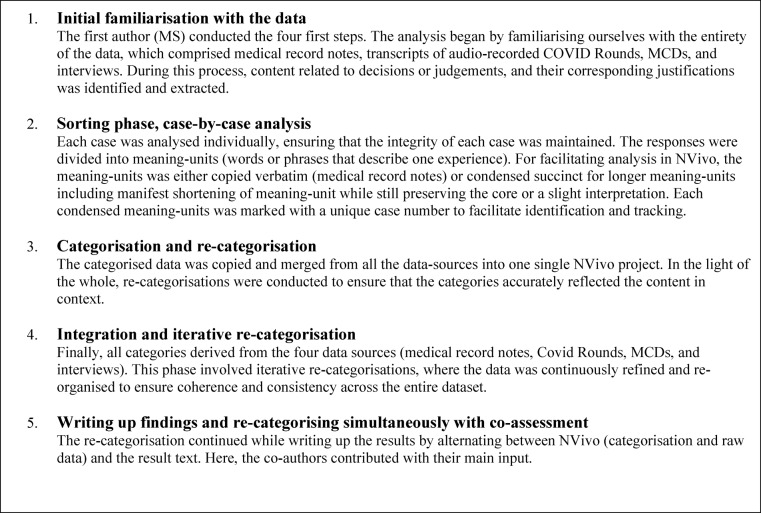
The qualitative analysis steps, with support from software program QSR International, NVivo V.12 [32]

### Regression and correlation analyses

We conducted four multiple regression analyses, with ICU admission, documented decision, decided level-of-care and justified decision as dependent variables. First, a univariate regression analysis was conducted for each dependent variable with age, CFS, CCI, pandemic wave and sex as independent variables. We dichotomized patients into “old” (65–79 years) and “very old” (≥ 80 years) [1, 2]. Second, variables with *p*-values < 0.20 and low collinearity (variance inflation factor < 2.5 [33]) were entered into a multiple regression analysis, using the Backward Stepwise (Wald) command in SPSS Statistics, v.29.0. Variables with *p*-values < 0.05 in the multiple regression were kept in the final regression model. Nagelkerke’s *R*^2^ was calculated for the final model. We also investigated the correlation between age, CFS and CCI, using Spearman’s correlation coefficient as the three variables were not normally distributed (Table 5).

### Triangulation

We applied data source triangulation from the audio-recordings, interviews and the medical records. We sought convergence and divergence across data sources of the qualitative and quantitative findings [25].

## Results

We investigated level-of-care decisions for older patients with COVID-19 by reviewing the medical records of 329 patients and analysing discussions concerning 34 patients during ten COVID rounds and nine Moral Case Deliberations, with the participation of 89 clinicians (see Table 2). Level-of-care decisions were documented in the medical records of 239 patients (73%). Among these, 84 (35%) included documentation *in favour* of ICU-admission and 129 (54%) contained documentation *against* admission. For the remaining 26 patients (11%), the decision was either unclear—containing both arguments in favour and against ICU admission—or involved multiple decisions with varying combinations of yes/yes, yes/no, no/yes, or no/no, reflecting uncertainty (will be published elsewhere).

Of the 239 patients with documented decisions, 56% included explicit justifications for the decisions made. A minority (31%) of decisions *favouring* ICU admission were explicitly justified, whereas a majority (88%) of decisions *against* admission were accompanied by clear justifications (see Table 2). The quantification of the qualitative analysis across all data sources yielded a total of 1,155 justifications. Approximately half of these favoured ICU admission, one quarter opposed it, and the remainder pertained to expectancy or uncertainty.

Justifications *in favour* of ICU admission were predominantly derived from the COVID Rounds and MCDs. Only 9% of the 1,155 justifications (regarding 26 patients) were justifications in favour in the medical records. Figure 1 illustrates the proportion of text devoted to each category of justification and delineates the differences in coverage between COVID Rounds/MCDs and medical record notes.

**Table 2:** Sample: patients, data-collection and clinicians

Data sources:	COVID rounds	Moral Case Deliberations	Medical records
**Included patients**, *n*	25	9	329
Female/Male, *n*	13/12	3/6	130/199
Age, *median (range)*	70 (48**^**–93)	70 (56**^^**– 83)	76 (65–99)
Pandemic wave 1/2, *n*	25/0	3/6	145/184
**Audio-recordings**, *n*, *mean min (range)* **Interviews** *min*,* mean min (range)*	10, 22 (10–34) 4, 36 (19–53)	9, 63 (43–90) 4, 28 (15–40)	**-**
**Patients with a documented justification**, *n*	-	-	**135**
**Participating clinicians** *n/Median per occasion (range)*	38/4 (3–5)	51*/5 (4–26)	
Female/male/unknown, *n*	24/14/0	13/12/26*	
*Title*			
Consultant	15	12	
Specialist	7	7	
Registrar	7	11	
Unknown	9	21*	
*Specialty*			
Intensive care	15	38*	
Infection	4	9	
Other medical specialties**	10	4	
Unknown	9	0	

*Including MCD 11 on a weekly meeting for 26 intensivists

**Medicine, Haematology, Reumatology, Geriatrics, Primary care and one physiotherapist

^5 patients were < 65 years old, three missing, but described as old in discussions.

^^ 2 patients were < 65 years old.

### Decisions with explicit justifications

#### Descriptions of decisions

The medical record notes reflected varying levels of care. Decisions in favour of ICU admission were typically described as either “full treatment on ICU” or “ICU treatment with limitations”. In contrast, decisions against ICU admission were most phrased as “0 ICU, 0 CPR”. Other descriptions were vaguer, ranging from ward-based care with all possible treatment, to ward care with specified limitations, or simply “0 CPR” without further detail. For 16 patients (5%), a decision for palliative care was documented, of which four included explicit justifications.

“*To avoid exposing the patient to unnecessary suffering*,* the decision was justified with a palliative approach*” (Medical record 52).

### Descriptions of justifications

Justifications included descriptions of medical benefit, general condition/function, age, professional duty, the sense of whether it was “worth giving it a go,” and concerns about safety and security (Fig. 1; Table 3). These justifications varied across the data sources. In medical records, medical benefit, good general condition/function, and age were the most cited reasons in the medical records. In contrast, grit and professional duty were more prominent in the COVID Rounds and Moral Case deliberations (Fig. 1).

**Table 3:** Justifications in favour of and against ICU-admission, categorised as domains, categories and subcategories

		Quotations from the Medical Record (MR)	COVID rounds (CR) or Moral Case Deliberation (MCD)
**Domain 1**	**MEDICAL BENEFIT**		
**Category 1**	**Yes: Not-too-ill**		
Subcategory 1	Previously relatively healthy	Given the sparse background diseases (Medical Record (MR) 276)	*No physical illness that presents an obstacle* (COVID round (CR 5.1)
Subcat. 2	Stable or treatable	Palliative chemotherapy. Responded well to treatment with regression of metastatic burden (MR 305)	*Definitionally not severely ill yet*. (CR 7)
**Category 2**	**No: Too-ill**		
Subcat. 1	Multi-/comorbidities	… and severely affected by multiple serious diseases in at least three organ systems (MR 292 decision 2)	*Had his diabetes*,* his hypertension*,* and his chronic kidney failure*. So, he was carrying a heavy load. *(CR. 1.1)*
Subcat. 2	Non-beneficial	… due to the patient lacking the conditions to benefit from such care and treatment. (MR 276)	None
Subcat. 3	Bad survival prognosis	Just been discharged from geriatrics where she was treated for an abdominal abscess, remaining drain. Lost weight … doubtful if the patient can tolerate ICU. (MR 109 dec. 2)	*Neutropenic*,* impaired immune system*,* risk with ventilator*,* so preferably remain in the ward* (Interview ICU Cons*-CR 8)
**Domain 2**	**GENERAL CONDITION/FUNCTION**		
**Category 1**	**Yes: Good CONDITION/FUNCTION**		
Subcat. 1	Good functional status	… walks several kilometers with a cane or walker due to the amputations. (MR 287)	*To play 18 holes of golf while walking gives me an idea of the endurance and strength one has; an attempt to objectify i*t (ICU-spec*+ cons, MCD 15)
Subcat. 2	Independent	The patient reports that she lives alone without home care. She cleans, does laundry, cooks, and shops. (MR 250)	Lives in a house with wife, and they seemed to take care of the home largely by themselves. (MCD 14)
Subcat. 3	Active	Is active with dog walks and gardening. (MR 149)	*He still works; he has changed jobs now since he is 72 this year.* (Med.reg** CR 2.1)
Subcat. 4	Good cheer and vital	The patient is usually well despite their illness. (MR 305)	*He was 80 years old but very spry.* (Inf.cons***, MCD 15)
**Category 2**	**No: frail/POOR CONDITION**		
Subcat. 1	Expressed “frail”	Given the patient’s deteriorated general condition and significant need for home help, she can be considered CFS 7–8. (MR 206)	*Yes*,* she is [CFS] 7–8 somewhere around there*,* she can’t manage much on her own*,* so at least 7 points. And more than 5*,* one thinks that respiratory treatment is not so beneficial.* (Med spec**. CR 3.1)
Subcat. 2	Dependent	The patient needs help with dressing, especially with trousers. The couple hasn’t had the energy to vacuum since last summer. (MR 190)	*He cannot manage ADL*,* and if we then lower his functional level further*,* as is done with intensive care*,* he will slip further down… In my opinion*,* it almost feels impossible to be able to recover intensive care*,* to bounce back to the same level.* (Inf. Spec***, MCD 17)
Subcat. 3	Inactive, immobile	None	
Subcat. 4	Run down	Considering biological age. (MR 42) Considering poor condition before illness. (MR 59 Dec. 2)	… *suffered both an elbow fracture and a pelvic fracture in Feb*,* went home in a wheelchair … had fallen indoors and fractured … clearly*,* she has become weaker from it … hmm*,* maybe not so active. (Med cons + ICU-cons.CR 3.3)* *He didn’t look like someone who read*,* um “Life and Health” regularly*,* he looked quite deteriorated*. (Inf-spec., MCD 17)
Subcat. 5	Low chance of recovery	Difficulty recovering from a hip fracture makes it uncertain whether she would manage to recover from intensive care. (MR 281)	*It might have been possible to keep her alive*,* but then she would die two weeks later because her body is worn out. There is no rehabilitation potential.* (ICU-cons: interview after MCD Weekly meeting)
Subcat. 6	No margins	None	*Not strong*,* muscle strong … he doesn’t have much reserve*. (Med reg CR 9)
**Domain 3**	**Professional duty**		
**Category 1**	**Yes: Benefit of doubt**		
Subcat. 1	Give a chance due to uncertainty	They don’t feel they have enough background to say no to intensive care and ventilation. (MR 139)	*Not being able to deny him that chance, would have been too painful.* (MCD 12)
Subcat. 2	Duty to keep trying	None	*He can’t die here. It doesn’t feel right that he should die here: If he does die, we must have done a little more.* (Med cons MCD 13)
Subcat. 3	Uncertain about patient’s wishes	Bringing up the issue of CPR and intensive care; the patient has not considered this. (MR 97)	[see text]
**Category 2**	**No: Do no harm**		
Subcat. 1	Impede unworthy suffering	Long-term respiratory care in ICU, not likely to be successful and would result in low quality of life after a long rehabilitation period. Therefore, ICU care would only prolong suffering.(MR 133 Dec. 2)	*Considering the long rehabilitation, possibly a lot of suffering, and still an outcome that ends in death. Then it’s not a good thing*. (Med ger^., MCD 18)
Subcat. 2	Not expose to torture	None	All we have done then is to torment a person for two weeks. Then we have done nothing good. (ICU-cons, MCD 11)
Subcat. 3	Ventilator treatment is hazardous	None	One loses a lot of muscle mass and neurological functions. Especially those who are on a ventilator for a long time, many can barely move when they come off it. (Inf.Spec: MCD 17)
**Domain 4**	**AGE**		
**Category 1**	**Yes: Not-too-old**		
Subcat. 1	Give a chance due to age	None	*From what I remember, there were ICU-beds. I thought considering her chronological age, then you give her a chance*. (ICU-cons, MCD 16)
**Category 2**	**No: High age**	**Category 2**	**No: High age**
Subcat. 1	“Considering age”, “and age”	Considering the patient’s advanced age, not suitable for ICU. (MR 31)	*Very old* [age 73] (Med. Cons. CR5.2)[see text]
**Domain 5**	**WORTH GIVING IT A GO OR NOT**		
**Category 1**	**YES, GRIT AND WILL TO LIVE**		
Subcat. 1	Fighter	None	*He knew he would die from his cancer, but he described it as, “I’m a fighter, and I’ll take this too. ICU-spec: he conveyed very strongly that there was no doubt he would fix this.* (ICU-cons+ICU-reg MCD 12)
Subcat. 2	Determination	The patient himself is interested in ventilator care and wants every chance to survive. (MR 305)	*If she were to be on a ventilator for two to three weeks, she would still be motivated to come back*. Primary care registrar: *She’ll be up before anyone asks for it.* (ICU-spec, CR 1.5)
Subcat. 3	Joy of life/ a good life	None	[See text]* + He lived a good life and that it was worth full effort*. (ICU-spec MCD 15)
**Category 2**	**NO: LACK OF WILL AND COPING**		
Subcat. 1	Not making an effort	None	*Not been someone who has fought very hard to keep going and stay healthy* (Inf reg MCD 16) *She continues to smoke and she knows what it’s like and still chooses to live the way she does. Then I feel it is doubtful *(ICU-cons., CR 1.6)
Subcat. 2	Conveyed not able to bear it	Pat is scared, quite adequately, that IVA care is a struggle and that he won’t get well. (MR 2b)	*"She had said when the ambulance picked her up, 'I don't want to do this anymore, I will never come home again.'".* (CR 3.1)
Subcat. 3	Conveyed limits of treatment	Discussed treatment limitations with the patient. The patient does not want to be put on a ventilator. (MR 28)	*What speaks against it was … and where she herself has refused treatment. But we don’t know why *(ICU-spec, CR 6.1)
**Domain 6**	**SENSE OF SAFETY AND SECURITY**		
**Category 1**	**YES, SAFETY FOR CONCERNED**		
Subcat.1	Better care in ICU	None	*… be liberal with it [to offer ICU-care]. *Ethicist:* do you think that it is something good to offer? It depends on what you are looking for, but I would say that it is something good*. (Ger.-reg^^ MCD 18)
Subcat. 2	Family care	None	*That we on the ward should not have to say no … just that step of saying no, he is not allowed to come to IVA – feels very harsh towards family ... then I can convey the feelings that we have done all.* (Inf. Cons, MCD 12)
Subcat. 3	Relieve staff’s burden and uncertainty	None	[See text]
Subcat. 4	Joint doctor consultation	None	[See text]
**Category 2**	**NO, SECURITY FROM TRUST**		
Subcat. 1	Leaning on previous decisions	None	*They called me and ask about ICU-care. Then I read the medical record and the ICU-cons X note … I trust him very much, who has made a thorough judgment with very reasonable positions. I thought we were very much in agreement. (ICU cons 1 + 2, MCD 11)*
Subcat. 2	Leaning on joint consultation	Brought up for discussion again with patient who cannot handle such a discussion due to confusion. Conversation with the sons and intensivist on call. Overall, the judgment is that ICU- care is not deemed to be able to benefit him. (MR 236)	[See text]

* Intensive care consultant/registrar, ** Medical registrar/consultant, *** Infection consultant/registrar, ^Geriatric registrar, ^^Hematology consultant

### Justificationsin favour andagainst ICU-admission in different data-sources

Justification of medical benefit weighed the severity of illness in *favour of* or *against* admission. General condition/function referred to being in good condition versus being frail. Age considerations included the notion of not being too old versus having a high age. Professional duty balanced the obligation of giving the patient a chance versus avoiding harm. Safety and security were only brought up in COVID Rounds and MCDs. ICU admission was seen as ensuring safety, while security was felt through trust in peers as an argument against admission.

### Justifications in favour of ICU-admission

In the medical record notes, the primary justification *in favour* of ICU-treatment was a good general condition/function. This accounted for 30% of the text coverage (Fig. 1) and justifications regarding 20 of the 26 patients with justifications *in favour* of admission. This reflected an image of patients as independent, active, and vital, with a good functional status. Being not so ill accounted for 18% described as previously being relatively healthy or having a stable or treatable medical condition (Table 3).

To give the benefit of the doubt was conveyed across the data sources (18% medical records, 26% Covid Rounds/MCDs), suggesting a duty to continue efforts or giving a chance despite uncertainties. One explicit uncertainty was about patient’s wishes, motivating continued efforts: “*Difficult when talking to a patient who is quite ill … Does she understand the implications of it* [intensive care]?” (MCD 16).

“Worth giving it a go” was an expression frequently used by intensivists during COVID-rounds and MCDs. Worth seemed to mean to benefit from ICU and during reflection grit emerged as a phenomenon. It manifested in expressions like “*had the forehead bones*” [in Swedish pannben, connected to elite sport], “*fighter*”, “*determined*”, “*have spark*”, “*a fighting spirit*”, “*gunpowder old chap*”, embodying a mental strength of being strong, not surrendering and able to bear blows.,” This was described as necessary for coping on ICU and in the recovery phase (Table 3).

There was also conveyed a respect for patient’s determination to survive and being struck by patient’s will-to-live, mostly described as living a good life or experiencing joy in life despite a pessimistic prognosis. “*He had a joie de vivre*,* an ability to take matters into his own hands*.” (ICU-spec.MCD 13).

One explicit note about patient’s wishes was found in the medical record “*very strong wish from patient and family about maximum treatment.”* (MR 305).

In COVID Rounds and MCDs, a sense of safety for the concerned was linked to ICU-care, offering enhanced care and monitoring, providing family support and alleviating the burden on ward “*Although it feels safest for me if the patient is on ICU. That is the ward perspective. Everything is safe on ICU*.” (Med spec, MCD11).

There was also a reassurance of joint doctor consultation: *“We agreed yes. we agreed that we can give a chance*,* but still being extremely uncertain how he will manage (ICU-cons) … doctors with clinical experience who lean in the same direction. It says a lot*,* the same feeling*,* the gut feeling.”* (Med. reg, MCD 13).

#### Justifications against ICU-admission

The primary justification *against* ICU-admission in the medical records was being too-ill, which accounted for 58% of the notes (Fig. 1). This included having comorbidities, having a bad survival prognosis and ICU-care being judged as non-beneficial. Too-ill was followed by frail or in poor condition, described as dependent, inactive, immobile, run down and with no margins and low chance of recovery (Table 3). High age was the justification for 47 patients but shortly stated, such as “*considering age*” (“*high biological age*” was categorised as being frail/in poor condition). The patients described as being too old had a median age of 84 (range 70–97).

During COVID Rounds and MCDs, justifications of being too-ill also prevailed (34%), but was followed closely by do no harm (26%) and frailty (20%) (Fig. 1). The rationale to do no harm included avoiding unworthy suffering, not subjecting patients to torture if not benefitting from ICU and recognising the risks of ventilator treatment (Table 3). An age of 70 was considered acceptable in favour of ICU-admission, but being beyond 80 was not, and at 90 there was a view that it was natural to die.

*“Ninety feels like*,* if you’ve had a good life and you’re spry and healthy … if you die suddenly*,* then it’s expected … And better than dragging to the ICU and tormenting the person*.” (ICU-cons + ICU-reg MCD 19).

Other justifications included a lack of will/coping, referring to patients not making an effort, expressing an inability to endure, or conveying treatment limitations (Table 3). A security from trust for the decision to not admit the patient was expressed from leaning on previous decisions and joint consultation.

*“I was very pleased when I left. I thought it was a good discussion. …I thought the same as ICU-specialist … it was done well even with the patient*,* so I left feeling pleased. I didn’t think it was obvious how to do it … though there was a discussion and conclusion of the whole*.” (Hematologic cons, MCD 19).

### Justifications explicitly linked to the pandemic

Few justifications were directly related to the pandemic (Table 4). Explicit COVID related justifications comprised 126 (11% of 1155) of the condensed meaning units. Not being too-ill implied having a physiological reserve to fight COVID and looking better than objective parameters (Table 4). Giving the benefit of the doubt was motivated by uncertainty of the course of COVID-infection or whether surviving high-flow treatment on ward and compensating for iatrogenic spread of the infection.

**Table 4:** Explicit COVID justifications in favour of and against ICU-admission categorised as domains, categories and subcategories with quotes in medical record (MR) and COVID-rounds (CR)/Moral case deliberation (MCD)

Quotes:	Medical Record (MR)		COVID-rounds (CR) or Moral Case Deliberation (MCD)			
**MEDICAL BENEFIT** **Yes: Not-too-ill**						
Stable or treatable	None			*Do not believe in higher mortality from COVID than from myeloma (MCD 14).*		
**No: Too-ill**						
Will not survive COVID	The patient’s overall comorbidity and current severe COVID pneumonia with expected progression.(MR 237)					None
Risk condition COVIDd	Likely not reasonable considering the overweight. (MR 183 2b)			[see text]		
**GENERAL CONDITION/FUNCTION**						
**Yes: Good**						
Physiological reserve	None None	*He had the physical capacity with muscle strength to be able to recover respiratory muscle-wise if he managed to overcome the COVID infection*. (ICU-cons MCD 12) *Especially with COVID*,* that the patients are so respiratory affected and do not look so sick … more difficult for the relatives who do not really understand how ill the patient is*. (Inf. Cons. MCD 15)				
Looks better than parameters						
**No: frail/POOR CONDITION**						
Low functional level	None	*Underlying functional level*,* so if she becomes critically ill … that is*,* requires intensive care due to COVID*,* we do not believe that she would benefit from being placed on a ventilator.* (ICU-Cons, 11 MCD)				
**Professional duty**						
**Yes: Benefit of doubt**						
Give a chance due to uncertainty	None	*We don’t know enough of the covid infection*. (MCD 11)				
Will not survive on ward	None	*90% oxygen on high flow. She was having a tough time. … got worse and then deteriorated quickly during the night.* [Ethicist cites MR]: *“*will certainly lead to problems even on a ventilator.*” How did you perceive this?* ICU-cons: *I honestly don’t know; I don’t think I read it. Since she was so bad when I arrived*,* I just took her in. I didn’t perceive that there had been any discussion about the level-of- care.* (MCD 16)				
Iatrogenic compensation	None	[see text]				
**No: Do no harm**						
Threat to dignity	None	*Undignified end in the COVID ICU if not surviving*,* without farewells with loved ones.* (MCD 13)				
Avoid chaos on Covid-ICU	None	[see text]				
**AGE**						
**Yes: Not-too-old**						
Give a chance due to age	None	*90% of COVID patients at the same age [65] benefit from intensive care*. (Inf-spec MCD 17)				
**No: High age**						
Too old to survive long ventilator time	None	*It’s partly about age and the conditions to manage 14*,*10 days in ventilator maybe*,* I don’t think she …* (ICU-Cons CR 3.3)				
Age as a risk factor	None	*…not young*,* 70*,* So maybe that’s a negative thing for COVID.* (CR 1.1) *Age against her*,* 81 years*,* considerable age cons COVID*. (MCD 15)				
**WORTH GIVING IT A GO OR NOT**						
**YES**,** GRIT AND WILL TO LIVE**						
Respect for patient’s determination	None	*He was awake and lucid … there was no discussion on his part*,* I was tough*,* trying to explain not only that it’s painful but also that you might die without being able to see your relatives. Then he said*,* “Let’s go!*” (ICU-spec, MCD 12)				
**NO: LACK OF WILL AND COPING**						
Conveyed can’t bear	None	[see text]				
Not compliant	*She is demented and*,* on the ward*,* has not accepted oxygen therapy*,* removing it all the time*. (MR 14)				None

*“Got COVID in connection with the fistula procedure … maybe contracted a secondary illness because we were there and did something that could very well influence to make the decision in a more interventionist direction. To try to compensate for that.”* (ICU-cons CR 1.8).

Respecting the patient’s wishes was a justification to the extent the patient was considered lucid and able to reason despite severe respiratory insufficiency. For justifications against admission, medical factors such as a poor survival prognosis were described, with obesity, hear condition and cancer frequently mentioned as risk factors.

*“Long discussion*,* it bothered me that he wouldn’t be able to get his cancer treatment … which was very bad for him since we had information that there was a very high mortality rate on ventilator and COVID. And to then put someone with metastatic cancer.”* (Hem.conc, MCD 12).

Additionally, concerns about not harming the patient by exposing them to the chaotic environment of a COVID ICU were noted.

*“Schizosyndrome*,* must be hell on earth to end up in COVID-ICU*,* like a Turkish bazaar with people shouting at each other behind these masks… very high noise level*,* all the machines beeping and running*,* no privacy*,* no peace and quiet*,* it’s terrible. And then people walking around in gas masks with strange clothes.”* (ICU-cons CR 10).

#### Influencing factors regarding ICU-admission in medical records

Overall, there was no substantial collinearity in the univariate regression analyses. In the multiple regression analyses, age was significantly associated with all four outcomes: ICU admission, documented decision, decided level-of-care and justified decision (Tables 5 and 6). Age ≥ 80 was significantly associated with lower odds of being admitted to ICU in the multiple regression. Age ≥ 80 and CFS ≥ 5 was associated with higher odds of having a documented decision and the second pandemic wave was associated with lower odds. Age ≥ 80 and CFS ≥ 5 was associated with higher odds of having a non-admission decision, but only age ≥ 80 was associated with higher odds of having an explicitly justified decision. Age correlated moderately with CFS (*r* = 0.45, *p* < 0.001) and CCI (*r* = 0.36, *p* < 0.001) (Table 6).

**Table 5:** Influencing factors being admitted to ICU (*n* = 329)

Influencing factors		Age, years		CFS		CCI		Pandemic wave		Sex	
Was the patient admitted to ICU?		65–79	80–99	1–4	5–9	3	4–12	Wave 1	Wave 2	Male	Female
		*n* = 206	*n* = 123	*n* = 148	*n* = 139	*n* = 21	*n* = 307	*n* = 145	*n* = 184	*n* = 199	*n* = 130
**Yes**,** n (%)**	*n* = 42 (13)	40 (19)	2 (2)	28 (19)	8 (6)	3 (14)	39 (13)	20 (14)	22 (12)	29 (15)	13 (10)
**No**,** n (%)**	*n =* 287 (87)	166 (81)	121 (98)	120(81)	131(94)	18(86)	268(87)	125(86)	162 (88)	170 (85)	117 (90)
**Univariate regression**	*OR (95% CI)*	1 (ref)	0.07 (0.02; 0.29)	1 (ref)	0.3 (0.1; 0.6)	1 (ref)	0.9 (0.2; 3.1)	1 (ref)	0.8 (0.4; 1.6)	1 (ref)	0.7 (0.3; 1.3)
	*p*-value		**< 0.001** ^a^		**0.001** ^a^		0.83		0.62		0.23
**Multiple regression**	*OR (95% CI)*	1 (ref)	0.03 (0.005; 0.25)	1 (ref)	NS^b^						
	*p*-value		**< 0.001**								

*CCI* Age-adjusted Charlson Comorbidity Index; *CFS* Clinical Frailty Scale, *CI* Confidence interval, *OR* Odds ratio

^a^Included in multiple logistic regression analysis. ^b^Not statistically significant (p > 0.05) in multiple logistic regression analysis. Nagelkerke’s R^2^ was 19.6%.

#### Corrobated evidence of impact of chronological age

Quantitative analysis identified age ≥ 80 as significantly associated with non-admission to ICU and qualitative data from all data sources revealed high age as a recurrent justification *against* admission. The qualitative and quantitative data converged and informed each other. In the qualitative findings of high age, ‘Considering age’, ‘and age’ in the medical records (numerous mentioned) and description of ‘very old’ in the COVID Rounds, MCDs and interviews converged with the quantitative findings regarding age ≥ 80 significantly associated with lower odds of being admitted to ICU. This provides corroborated evidence for impact of chronological age on ICU-Decision-making.

**Table 6:** Factors influencing (1) whether the decision on level-of-care was documented (*n* = 329); (2) whether the documented decision was justified (*n* = 239); and (3) whether the documented decision was a yes or no to ICU-care (*n* = 213; 26 patients were excluded because judgements contained expactance or uncertainty)

**Influencing factors**:		**Age**,** years**		**CFS**		**CCI**		**Pandemic wave**		**Sex**	
**1) Was the decision documented?**		***65–79***	***80–99***	***1–4***	***5–9***	***3***	***4–12***	***Wave 1***	***Wave 2***	***Male***	***Female***
		*n* = 206	*n* = 123	*n* = 148	*n* = 139	*n* = 21	*n* = 307	*n* = 145	*n* = 184	*n* = 199	*n* = 130
**Yes**,** n (%)**	*n* = 239 (73)	135 (66)	104 (85)	101 (68)	121 (87)	9 (43)	229 (75)	116 (80)	123 (67)	143 (72)	96 (74)
**No**,** n (%)**	*n* = 90 (27)	71 (34)	19 (15)	47 (32)	18 (13)	12 (57)	78 (25)	29 (20)	61 (33)	56 (28)	34 (26)
**Univariate regression**	OR (95% CI)	1 (ref)	2.9 (1.6; 5.1)	1 (ref)	3.1 (1.7; 5.7)	1 (ref)	3.9 (1.6; 9.6)	1 (ref)	0.5 (0.3;0.8)	1 (ref)	1.1 (0.7; 1.8)
	p-value		**< 0.001** ^a^		**< 0.001** ^a^		**0.003** ^a^		**0.008** ^a^		0.69
**Multiple regression** ^**c**^	OR (95% CI)	1 (ref)	2.1 (1.1; 4.1)	1 (ref)	2.5 (1.4; 4.8)	1 (ref)	NS ^b^	1 (ref)	0.5 (0.2; 0.8)		
	p-value		**0.032**		**0.004**		NS ^b^		**0.011**		

**Influencing factors**:		**Age**,** years**		**CFS**		**CCI**		**Pandemic wave**		**Sex**	
**2) Was the documented decision justified?**		***65–79***	***80–99***	***1–4***	***5–9***	***3***	***4–12***	***Wave 1***	***Wave 2***	***Male***	***Female***
		*n* = 135	*n* = 104	*n* = 101	*n* = 121	*n* = 9	*n* = 229	*n* = 116	*n* = 123	*n* = 143	*n* = 96
**Yes**,** n (%)**	*n* = 135 (56)	62 (46)	73 (70)	53 (52)	82 (68)	2 (22)	132 (58)	69 (59)	66 (54)	78 (55)	57 (59)
**No**,** n (%)**	*n* = 104 (44)	73 (54)	31 (30)	48 (48)	39 (32)	7 (78)	97 (42)	47 (41)	57 (46)	65 (45)	39 (41)
**Univariate regression**	OR (95% CI)	1 (ref)	2.8 (1.6; 4.8)	1 (ref)	1.9 (1.1; 3.3)	1 (ref)	4.8 (1.0; 23.4)	1 (ref)	0.8 (0.5; 1.3)	1 (ref)	1.2 (0.7; 2.1)
	p-value		**< 0.001** ^a^		**0.021** ^a^		0.054 ^a^		0.36		0.46
**Multiple regression** ^**d**^	OR (95% CI)		2.6 (1.5; 4.6)	1 (ref)	NS^b^	1 (ref)	NS^b^				
	p-value		**0.001**								

**Influencing factors**:		**Age**,** years**		**CFS**		**CCI**		**Pandemic wave**		**Sex**	
**3) Was the documented decision a yes to ICU?**		***65–79***	***80–99***	***1–4***	***5–9***	***3***	***4–12***	***Wave 1***	***Wave 2***	***Male***	***Female***
		*n* = 120	*n* = 93	*n* = 85	*n* = 111	*n* = 9	*n* = 203	*n* = 106	*n* = 107	*n* = 125	*n* = 88
**Yes**,** n (%)**	*n* = 84 (39)	70 (58)	14 (15)	58 (68)	12 (11)	8 (89)	76 (37)	42 (40)	42 (39)	56 (45)	28 (32)
**No**,** n (%)**	*n* = 129 (61)	50 (42)	79 (85)	27 (32)	99 (89)	1 (11)	127 (63)	64 (60)	65 (61)	69 (55)	60 (68)
**Univariate regression**	OR (95% CI)	1 (ref)	0.1 (0.06; 0.2)	1 (ref)	0.06 (0.02; 0.1)	1 (ref)	0.07 (0.01; 0.6)	1 (ref)	1.0 (0.6; 1.7)	1 (ref)	0.6 (0.3; 1.0)
	p-value		**< 0.001** ^a^		**< 0.001** ^a^		**0.015** ^a^		0.96		0.057 ^a^
**Multiple regression** ^**e**^	OR (95% CI)	1 (ref)	0.2 (0.06; 0.4)	1 (ref)	0.06 (0.03; 0.1)	1 (ref)	NS ^b^			1 (ref)	NS^b^
	p-value		**< 0.001**		**< 0.001**						

*CCI* Charlson Comorbidity Index, *CFS* Clinical Fraility Scale, *CI* Confidence interval, *OR* Odds ratio, *p* probability value.

^a^Included in multiple logistic regression analysis

^b^Not statistically significant (*p* > 0.05) in multiple logistic regression analysis

^c^ Nagelkerke’s R^2^ was 13.3%. ^d^ Nagelkerke’s R^2^ was 6.7%. ^e^ Nagelkerke’s R^2^ was 52.6%

## Discussion

In our study, few justifications were explicitly linked to the pandemic and the findings echo those of pre-COVID studies [4–7, 28, 34, 35], suggesting that we have captured the essence of ordinary clinical decision-making. This indicates that we can use the pandemic context merely as a lens through which to explore decision-making in situations involving difficult choices. For instance, resource shortages were scarcely mentioned; rather, reflections centred on being spared from a lack of ICU beds. This aligns with findings from a British pre-COVID project in which the first author participated [4–7, 28, 34, 35]. In fact, in the Swedish county studied, ICU capacity was significantly expanded from 14 to 38 beds. The head of the ICU-department reported afterwards that there was no need to prioritise patients according to the Swedish guidelines for ICU triage [19]. Instead, ordinary decision-making was applied, that is, determining who should receive care based on patient factors irrespective of prioritisation and resource limitations [36].

In the medical records we mostly found documented decisions and explicit justifications *against* admission, but the qualitative data complements the findings with numerous and rich justifications *in favour* of ICU-admission. The integration of qualitative and quantitative findings provides corroborated evidence that high chronological age is a key factor against ICU admission.

In the following discussion, we will focus on the aspects of age and grit against the background of the principles *beneficence*,* nonmaleficence*,* autonomy*, and *justice* [37] as well as the ethical principles underpinning Swedish healthcare prioritisation legislations, that is, *human dignity*, *needs and solidarity*, and *cost-effectiveness* [38].

### Should age matter?

Firstly, distinguishing between chronological and biological age is crucial. While advanced chronological age often coincides with frailty and comorbidities [7], our study found only a moderate correlation between the two. In other words, patients with a high chronological age may or may not have a high biological age. Notably, chronological age remained the sole significant factor associated with not being admitted to ICU, indicating that clinicians already take chronological age into account in their Decision-making. As will be discussed later, chronological age also remains directly relevant from an equity standpoint—particularly if we hold that society should allocate resources to ensure all citizens the opportunity for an equally good and lengthy life.

Studies confirm that patients aged ≥ 80 have higher ICU mortality and poor recovery, supporting the argument that ICU-care for older patients may be more harmful than beneficial [1, 2, 39]. In our study, ‘do no harm’ was a common justification for non-admission, linked to the principle of *non-maleficence* [37]. This indicates that ICU-care is a source of suffering, loss of dignity, and even torture supported in previous studies [4, 28]. We argue that *non-maleficence* should take precedence over *autonomy*, as patients, referring doctors, and the public often underestimate the suffering ICU care entails.

The principle of justice motivates the use of chronological age in healthcare resource allocation. Justice may require rationing benefits for one group to prioritise others. Distributive justice theories, including utilitarianism, prioritarianism, and egalitarianism, argue for a lifetime perspective in resource allocation [40, 41]. This implies that a distribution is effective (utilitarianism) or fair (prioritarianism/egalitarianism) over the course of whole lives. This means that younger patients generally are prioritised over older ones, who stand to gain less (effectiveness) or have already had a greater share of life and health (fairness). There are few theoretically substantiated views where it is argued that age should *not* be considered (see for example Harris 1985 [42]). What we do find, are more pluralistic views on how to relate to age, or more generally, time perspectives (see [40, 43, 44]). However, all these views will allow chronological age to make some difference in the priority of resources. The main criticism of this approach is ageism or sub-optimal treatment [2, 40, 45]. This might, in turn be countered by the argument that not taking age into account would constitute ageism towards the young. This is contrasted by the Swedish legal priority-setting principles guiding healthcare —*human dignity*, *needs and solidarity*, and *cost-effectiveness* [38]. The *Human Dignity* principle states that chronological age, per se, should not be a priority setting criterion, and that general age limits for receiving care ought to be avoided. Nevertheless, consideration of biological age is permissible, albeit the legislation remains ambiguous regarding its precise application [38].This was one reason why the Swedish COVID guidelines [46] sparked controversy. The utilisation of life expectancy, derived from biological age, as a criterion sparked public debate on allegations of age discrimination—primarily framed as a legal issue, given the absence of explicit legislative endorsement—rather than as an ethical concern [21, 47]. However, this legal objection was not universally prevailing, and there was also significant support within the discourse for incorporating biological age in clinical decision-making.

Norwegian legislation, on the other hand, allows for indirect consideration of chronological age by prioritising the distribution of life-years [48], which aligns with ICU prioritisation literature excluding patients “*with a fuller lifecycle”* [2]. 2This indicates acceptance of allowing chronological age to indirectly play a role in assessment of effectiveness in a balance between costs and benefits [38]. In comparison with other publicly funded healthcare systems, chronological age is not used as a sole criterion; rather, age is considered alongside frailty. For example, Canada bases its prioritisation on short-term mortality risk, as outlined in the Ontario Adult Critical Care Emergency Standard of Care, which includes factors such as age over 65 combined with a Clinical Frailty Scale (CFS) score greater than 7 [49–51]. Similarly, Switzerland adopts a comparable approach in its guidelines, emphasising “not age, but age-related frailty” [52].

However, recently the consideration of life expectancy loss was introduced as a criterion for assessing severity, in line with the principle of *need and solidarity*. This, in turn, indirectly allows chronological age to influence priority setting [53]. The principle of *need and solidarity* are connected to distributive justice but adds a shared moral responsibility towards the most vulnerable members of society, with a particular focus on equality [38]. Solidarity is deeply rooted in the Nordic societies, where collective needs are emphasised over the American focus on individual autonomy [54]. In ICU prioritisation, defining ‘needs’ is critical. The legal proposition underlying Swedish legislation [38] defines ‘needs’ as the severity of a condition and the potential benefit of treatment, in accordance with theoretical analyses [38, 55]. As previously mentioned, the higher ICU mortality and poorer recovery rates among older patients [1, 2, 39] suggest that they may not *need* ICU-care.

Not *needing* ICU-care does not imply that the patient should not receive any care, but points to the *need* for palliative care. This aligns with *beneficence*, with a focus on quality-of-life and a palliative approach over life extension at the cost of suffering. References to palliative care was notably absent as a criterion in decision-making in the current study and other studies have also noted this [4], though it has been advocated as an important alternative [8]. Palliative care should be given high priority, as it focuses on the care of vulnerable individuals. Palliative care may indeed involve intermediate care [2, 4] for instance, postoperatively, provided the goal of treatment is to improve quality of life but, nota bene, no ventilator treatment.

### Should grit matter?

In our study, grit emerged as a justification *in favour* of ICU admission, particularly during MCDs. Judgements of will-to-live, reflected in having a good life did appear, but references to a mental strength to endure ICU care and recover dominated. Grit has been defined as persistence despite setbacks, often accompanied by positive thoughts [56]. Grit is widely recognised in elite sports, but is more commonly associated with education [56] and work life [56, 57] in research. Notably, we identified a single study in the field of psychiatric care, utilising a grit scale, suggesting its role as a resilience factor in recovery [58]. Factors somewhat related to grit; “state of mind” [7] and “coping strategies and life values“ [2, 59] have been highlighted in previous ICU studies. Interestingly, in our study, grit seemed more important than respect for autonomy, even though COVID-19 patients, despite severe respiratory insufficiency, were found to be lucid.

Could grit be an acceptable factor *in favour of ICU-admission*? This raises a potential conflict between the principles of *beneficence*, *need and solidarity*, and *cost-effectiveness*, on one hand, and *human dignity*, on the other. On one hand, grit may be linked to potential benefits from ICU care, as doctors in our study emphasised the importance of both physical and mental strength for recovery, representing a strong argument grounded in beneficence. However, there is a lack of consensus on how to define and assess grit, and limited research on its relationship with ICU outcomes.

On the other hand, *human dignity* is at stake. In this context, autonomy holds limited value, as patients possess only negative rights—that is, the right to refuse treatment. There is a potential risk of injustice if autonomy is exercised to justify admission. We observed indications that patients demonstrating grit were more able to argue for and fight their admission. Patients with grit might wrongly be associated with a higher human worth, compared to patients perceived as lacking a strong will. Doctors did self-critically examining their positive feelings toward patients exhibiting grit during MCDs.

The Swedish *Human Dignity* principle explicitly bans considerations of social situation or standing, hence any preconceptions about what a good life in a wider sense is, should be avoided. Furthermore, grit opens the possibility of misjudgement. True grit might be manifested in subtle ways; for example, a timid patient with grit may not display it outwardly, leading to false negatives. Conversely, an extroverted patient without true grit may be mistakenly perceived as possessing it, resulting in false positives. Moreover, healthcare professionals may assess grit based on perceived quality of life, potentially equating functional disability and psycho-social problems with low quality of life [3, 60, 61]. In this view, external signs of a desire to fight are taken as evidence of life worth living, while someone not displaying such signs may be considered as having given up. Yet, we know that external behaviour is a poor indicator of a person’s internal values. Given all the above, the case for considering grit as a criterion in favour of ICU-admission becomes less strong before further exploration in research.

### Drawing normative conclusions from empirical data

Before addressing some limitations of the study, it is important to briefly consider the challenges involved in drawing normative conclusions from empirical data about how people actually reason or act. It is widely accepted in meta-ethics that one cannot derive an “ought” solely from an “is” without additional normative premises—a principle often referred to as Hume’s law or the is-ought problem [62]. A detailed exploration of this issue is beyond the scope of this paper, but it suffices to note that in the above we find support for using both age and grit as factors for admitting patient to ICU. However, there needs to be a normative analysis to support these empirical facts about people views or action patterns. This is found when it comes to age but not (yet) with respect to grit.

### Limitations of the study

Our findings may not be generalisable to other healthcare settings, but still, it may be transferable to Western European settings in ordinary clinical work. The use of multiple data-sources and mixed methods allowed us to capture both consistent patterns and notable discrepancies. It could be considered a limitation to merge results from COVID Rounds and Moral Case Deliberation, as they represent different formats — the former being a regular clinical round, and the latter a space for reflection. However, we were struck by the similarity of findings, particularly concerning grit. We believe this strengthens the robustness of our conclusions. Regarding MCD as a space for reflection, we were struck by the doctors’ candidness and willingness to engage in self-criticism. This openness encouraged us, as ethicists, to challenge their perspectives, ultimately enriching the data even further. The midst of the peak of the first wave was a chaotic time and we miss some demographic data from the COVID Rounds, but we believe this weakness is weighed up by this unique historical data.

## Conclusions

We have contributed with broad and rich knowledge into decision-making regarding levels of care. Our findings highlight the need to improve documentation in medical records. Documenting the justification for decisions regarding ICU admission ought to be mandatory for frail and elderly patients. Clear and well-reasoned documentation not only facilitates future re-evaluation of the decision but also provides understanding for the nurses caring for the patient, as well as for the patient and their family who may request access to medical records. It also helps other doctors who may see the patient at a later stage of care to understand the reasoning behind the decision.

Our findings highlight chronological age as a key factor, supported normatively by the ethical principles of non-maleficence and justice. Grounded in the Swedish legal priority-setting principle of Needs and Solidarity—which prioritises care only when there is potential benefit—our results suggest that very old patients may be better served by palliative rather than intensive care. We therefore advocate for a national (and potentially international) guiding policy decision on triage systems for very old patients in everyday care. Such a policy should be developed with courage and transparency, explicitly supporting a palliative approach for very old patients. Above all, the goal must be to ensure good care—not merely to ration resources.

We do find support for considering grit as a factor in ICU admission decisions primarily based on its association with potential benefit from ICU care and recovery. However, this raises important ethical concerns. Firstly, its application may conflict with the principle of Needs and Solidarity if grit does not correspond to benefit of ICU-care. Secondly, relying on grit risks undermining human dignity by implicitly linking a patient’s worth to their level of grit. While grit might indicate potential benefit from ICU care, equating it with human worth risks biased decision-making, particularly toward vulnerable patients based on subjective assessments of grit. Given that grit remains underexplored in ICU research, we advocate for caution before incorporating it as a decisive factor in ICU admission decisions, pending further empirical investigation.

## Data Availability

The SPSS file supporting the quantitative results reported in the article are available from the corresponding author on reasonable request. The data cannot be shared due to patient confidentiality and national laws. However, the syntax file for the quantitative analysis is available from the corresponding author on reasonable request. The qualitative data are not available, as individual privacy would be compromised, but quotes supporting the findings are presented in Tables 3 and 4.

## Abbreviations

ICU: Intensive Care Unit
MCD: Moral Case Deliberation

## Footnotes

1 Level-of-care in this study refers to level (home, primary care, hospital ward, ICU [21], and goal of care, i.e. survival or quality-of-life (palliative care) [2].

2 However, following an intense debate (including concerns about age discrimination), the Norwegian provisions were amended so that the previous severity criterion was retained. Severity of illness and priority setting in Norway - regjeringen.no﻿
